# Resident-Memory T Cells in Tissue-Restricted Immune Responses: For Better or Worse?

**DOI:** 10.3389/fimmu.2018.02827

**Published:** 2018-11-30

**Authors:** Karin Steinbach, Ilena Vincenti, Doron Merkler

**Affiliations:** ^1^Department of Pathology and Immunology, University of Geneva, Geneva, Switzerland; ^2^Division of Clinical Pathology, Geneva University Hospital, Geneva, Switzerland

**Keywords:** resident memory T cells, chronic, inflammation, infection, autoimmune

## Abstract

Tissue-resident-memory CD8+ T cells (T_RM_) have been described as a non-circulating memory T cell subset that persists at sites of previous infection. While T_RM_ in all non-lymphoid organs probably share a core signature differentiation pathway, certain aspects of their maintenance and effector functions may vary. It is well-established that T_RM_ provide long-lived protective immunity through immediate effector function and accelerated recruitment of circulating immune cells. Besides immune defense against pathogens, other immunological roles of T_RM_ are less well-studied. Likewise, evidence of a putative detrimental role of T_RM_ for inflammatory diseases is only beginning to emerge. In this review, we discuss the protective and harmful role of T_RM_ in organ-specific immunity and immunopathology as well as prospective implications for immunomodulatory therapy.

## Introduction

During an infection, our immune defense operates in a sensitive balance in which the eradication of an invading pathogen should take place efficiently with the least possible damage to the body's own structures. For this, different subsets of immune cells have evolved, which form several lines of defense and are equipped with different functional specializations. Various leukocyte subsets—from broadly acting innate immune cells to antigen-specific and specialized lymphocytes—act together to constitute a joint defense reaction against infectious intruders. CD8+ (so-called cytotoxic) T lymphocytes are essential executors of the adaptive immune system and are particularly specialized in eliminating aberrant cells that are either infected with an intracellular pathogen or of tumorous nature. Regional and functional specialization can also be observed among CD8+ T cells, especially among memory T cells that provide long-term protection against reinfection with a previously encountered pathogen (1). While central memory (CM) T cells home to secondary lymphoid organs (SLO) where they provide a stem cell-like pool of highly-proliferative antigen-specific memory T cells, effector memory (EM) T cells lack homing receptors for SLOs and patrol the body, charged with effector molecules (2, 3). In the last decade, a third memory T cell subset, referred to as resident-memory (RM) T cells, has emerged as an important guardian providing potent local immune surveillance at sites of previous infection, especially at barrier sites in the body (4, 5). T_RM_ procure superior protective immune memory in comparison to circulating memory T cells (6, 7) and presence of T_RM_ in tumors is associated with enhanced tumor control and survival (8). The generation and maintenance of this non-circulating, “sessile” immune subset is therefore the focus of intensive research efforts, for example with the aim of developing more potent vaccines (9). Conversely, more and more reports start to emerge linking the presence of T_RM_ with chronic inflammation and autoimmune diseases (10). Consequently, we need to deepen our understanding of T_RM_ biology in order to consider protective and possible harmful functions of T_RM_ into our strategies for new therapeutic approaches. There is currently a tendency to generalize the observed T_RM_ functions across different organs, although some reports suggest that besides sharing a common differentiation program, T_RM_ generation seems to be influenced by multiple factors and also adapt to the environment of their tissue of residence. In this review, we will focus on the presumed role of T_RM_ in protective immunity, chronic inflammation and organ-specific autoimmune diseases. In particular, we will place special emphasis on CD8+ T_RM_, as they are the best studied T_RM_ population so far. However, other resident lymphocyte populations have also been described. The latter include resident CD4+ memory T cells (11) and several resident invariant lymphocyte populations, such as liver NKT cells, gut-associated intraepithelial lymphocytes [including CD8αα T cells and (mucosal-associated invariant T) MAIT cells], and skin- and gut-resident memory γδ T cells (12–15). Moreover, resident innate lymphocyte (ILC) populations have been reported (16). Although we do not discuss these populations further in this review, some of our considerations might also apply to these cell subsets.

## T_RM_ generation and maintenance

The principal hallmark of bona fide T_RM_ is their long-term persistence in non-lymphoid tissues (NLT) as a stable memory T cell pool independent of input from circulating T cells. T_RM_ are often identified by a combination of surrogate markers (see Table 1), the most commonly used being CD69 and CD103, which are associated to their persisting and resident phenotype. Phenotypically, T_RM_ resemble a mixture of T_CM_ cells and effector T cells expressing markers associated with homeostatic proliferation and survival, such as Ki-67 and Bcl-2, and effector function, such as Granzyme B and co-inhibitory molecules (6, 32). Table 1 summarizes frequently used T_RM_ markers in mice and humans. However, a mere phenotypical identification without functional analysis might include circulating T cell subsets that can transiently express e.g., CD69 and CD103 (33, 34). In order to unequivocally identify T_RM_, besides phenotypical analysis, functional experiments assessing T_RM_ tissue egress, persistence, and their disequilibrium with peripheral T_CM_ and T_EM_ cells are usually performed (6, 35). T_RM_ demonstrate a strong disequilibrium (>90%) in parabionts (36, 37) and remain stable in numbers even when recruitment of circulating T cells to NLT is inhibited (20, 27). In most NLT, with exception of the liver (25), T_RM_ are anatomically separated from the blood and therefore not accessible to intravenously applied antibodies (32, 38, 39).

**Table 1:** Frequently used T_RM_ markers in mice and humans.

**Marker**	**Expression**	**Proposed function**	**Mouse**	**Human**
CD69	Almost all	Antagonisation of S1P1-mediated tissue egress	(4)	(17–19)
CD103	Subset *	Epithelial location via binding to E-Cadherin	(4)	(18)
CD44	All	Binding to hyaluronic acid	(20)
Bcl-2	Subset	Longevity	(21, 22)	(23)
CD49a	Subset	Binding to Collagen and Laminin, specialization of effector function	(4, 22)	(19, 24)
CD101	Subset	Inhibition of T cell activation and proliferation	(25)	(19)
GrB	All	Cytotoxicity	(21, 26)	(24)
CD127	Subset	Homeostatic proliferation	(27–29)	(30)
S1P1low	All	Low sensitivity to tissue egress signals	(31)	(19)
S1P5low	All	Low sensitivity to tissue egress signals	(22, 26)
CD62L low	All	Low sensitivity to tissue egress signals	(21, 4)	(19)
Ccr7low	All	Low sensitivity to tissue egress signals	(26)	(18)
CX3CR1low	Subset	Low sensitivity to tissue egress signals	(26)	(19)
KLRG1low	All	High memory potential	(22, 29)

* *Mucosal sites and skin. GrB, Granzyme B*.

In humans, T_RM_ and T_RM_-like cells are mostly identified in a descriptive manner based on the homology with mouse T_RM_ (17, 40) and by differential gene expression when compared to circulating memory T cell subsets (19, 24, 41). While functional analyses in humans are obviously more limited, studies in patients treated with immune-ablative regimens (42, 43), or transplantations of human tissue (44) indicate that human T_RM_ -like populations identified on this basis likely constitute a similarly stable persisting T cell pool. T_RM_-like populations in human NLT vastly outnumber T cells in circulation (17, 30, 45, 46), something that cannot be found in mice housed in a specific-pathogen free (SPF) environment, but in pet shop mice (47). Human T_RM_ will probably remain challenging to study, due to limited access to these cells and the lack of an *in vitro* culture system to this point. However, since not all aspects of human T_RM_ biology can be reproduced in SPF mice, a combined approach of mouse and human research will be instrumental to extend our knowledge about the role of T_RM_ in human health and disease.

### T_RM_ differentiation and maintenance program

T_RM_ mostly arise from CD127(IL7Rα)+KLRG1- memory precursor cells (22, 48, 49). Their differentiation into a long-term stably persisting and non-circulating cell population is based on two main requirements: the inhibition of tissue egress (residency) as well as longevity and/or homeostatic proliferation (maintenance). Once T cells have been recruited to the site of infection, T_RM_ precursor cells probably receive local signals from their future tissue of residence that guide the timely activation and inhibition of specific transcriptional programs. The most common mechanism is the upregulation of CD69, which antagonizes sphingosin-1-phosphate-receptor-1 (S1P1)-mediated tissue egress, and thereby confers early tissue retention until T_RM_ differentiation is complete (50–52). Most T_RM_ express CD69 constitutively and in the absence of CD69, T_RM_ generation in organs is strongly impaired (22). However, CD69 might be dispensable for long-term maintenance of fully-differentiated T_RM_, as has been described in the lung and the thymus (53, 54). Thus, temporary CD69 expression may be sufficient for T_RM_ generation and may explain the absence of CD69 expression on a subset of long-term persisting T_RM_ in the pancreas, salivary gland and female reproductive tract (37). Loss of S1P1, and potentially other tissue egress receptors, e.g., mediated by downregulation of the transcription factor KLF2 (31), together with expression of specific adhesion molecules, confers long-term tissue residency. Further, a combination of gene expression programs otherwise involved in the differentiation of both peripheral T_CM_ and effector T cells ensure maintenance of a stable population of T_RM_ by conserving proliferative capacity as well as acquisition of constitutive expression of effector molecules (49, 55). The transcription factors known to be involved in this process have been reviewed in detail recently (56, 57). T_RM_ and T_CM_ are probably generated from the same naive precursors (58), however, the gene expression profile of T_RM_ is clearly distinct from peripheral memory T cells in mice (22, 59) and in humans (19, 24, 41). In mice, particularly the expression of transcription factors Blimp1, Hobit, and Runx3 in T_RM_ precursors seems to be essential to acquire tissue residency (49, 59). For the maintenance of stable T_RM_ population, a combination of signals stimulating longevity and homeostatic proliferation seems to be necessary. Most T_RM_ express CD127 (IL7Rα), while expression of CD122 (IL2rβ), which can bind IL-2 as well as IL-15 when paired to CD132 (common γ chain, γc), seems to be more variable (22, 60). Previous studies have shown that IL-7 and IL-15-dependent longevity and homeostatic proliferation are maintaining T_CM_ by Stat5 signaling (61–63). Likewise, both cytokines have been implied to contribute to T_RM_ survival and maintenance (22, 64) and phosphorylation of Stat5 has been observed in a subset of brain T_RM_ (32). However, the sources providing homeostatic signals assuring T_RM_ long-term survival are so far still not completely known.

### Tissue-specific influences on T_RM_ differentiation and maintenance

**Figure 1:**
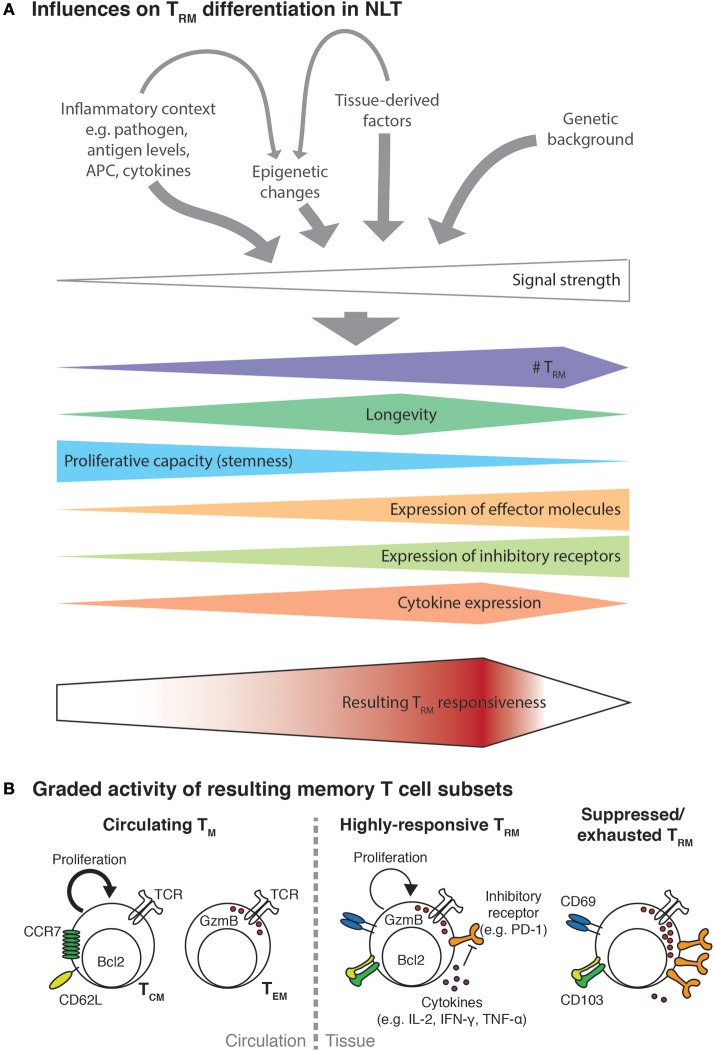
Multiple factors influence T_RM_ functionality. **(A)** Activated T cells recruited to NLT will encounter an inflammatory environment shaped by the nature and extent of infection. The encountered signals will consist of different cytokines (e.g., Il-2, IL-12, IL-15, type I and type II interferons) potentially in concert with varying levels of cognate antigen presented on professional APC and infected cells as well as tissue-derived trophic factors, metabolites or the microbiome. Probably additionally influenced by the genetic background of the infected individual, the strength of the resulting signal to activated T cells will direct their expansion and differentiation into T_RM_. In analogy to the signal strength model of CD8+ T cell differentiation (66), higher signal strength will result in higher T_RM_ numbers and be associated with more terminal differentiation, which manifests with progressive loss of proliferative capacity, acquisition of expression of effector molecules and increasing levels of inhibitory receptors. Encounter of very strong signals, such as during chronic infection, might lead to dysfunctional and exhausted T_RM_ and even to their elimination. The combined effect of all these factors will then determine the responsiveness of the resulting T_RM_ population to a secondary antigenic challenge or other inflammatory stimuli. **(B)** As a result of T cell activation and tissue-derived signal, circulating and resident memory T cells of different responsiveness will be generated. Circulating memory cells, namely central memory T cells (T_CM_) and effector memory T cells (T_EM_) show a delayed recruitment to the infected site. In addition, those cell subsets seem to specialize in either proliferative potential or immediate effector function. In contrast, a moderately strong T_RM_ differentiation signal will result in high numbers of highly-responsive T_RM_ that combine features of both T_CM_ and T_EM_ cells. Even though T_RM_ may express inhibitory receptors such as PD-1 to some degree, they can overcome this regulation e.g., due to their high expression of inflammatory cytokines (67). Highly-responsive T_RM_ can efficiently protect against re-infection but due to their low threshold for reactivation they could be prone to drive immunopathology or be involved in aberrant immune responses such as in allergies and autoimmune diseases. Alternatively, T_RM_ can be subject to regulation by regulatory T cells and other mechanisms, which may impair their longevity and/or induce a suppressed phenotype.

The gene expression program of T_RM_ generated in different tissues is largely overlapping (19, 22, 65, 59), but some variations of this program as well as particular requirements for T_RM_ differentiation seem to exist in different experimental settings, organs and even show inter-individual variability. A particular T_RM_ phenotype and its functional characteristics are thus likely to be due to pathogen- and tissue-specific cues as well as the genetic background of the host (see Figure 1A). Moreover, most T_RM_ markers are not homogeneously expressed in the whole resident population (18, 68), suggesting further specialization of a particular T_RM_ population into functional subsets—even if they have been generated by one definite infection and harbor the same antigen-specificity. Differential gene expression programs and surface receptor expression on putative T_RM_ subsets are likely to confer different tissue locations and functionality, as we will further discuss below. More detailed analysis, probably using single cell-based approaches will soon identify possible T_RM_ subsets on a phenotype and functional basis.

One of the major incongruities of T_RM_ differentiation in different organs is the dependency on local antigen expression. While T_RM_ in the gut, skin and some mucosae can be generated and maintained independently of local antigen presentation (69–71), expression of local antigen seems to be required for the generation of T_RM_ in the brain (29, 32). In theory, local antigen expression serves various purposes: In a very basic manner, local antigen expression will enhance recruitment and local expansion of T_RM_ precursor cells and thereby increase the resulting T_RM_ population (72). For some organs, local antigen expression might be strictly required for tissue entry of antigen-specific T cells, as suggested for the brain (73), and thereby be essential for T_RM_ generation. In general, however, inflammatory cues, such as certain cytokines and chemokines, seem to be sufficient to promote T_RM_ differentiation, such as evidenced by so-called “prime-and-pull” and “prime and trap” vaccination approaches, which efficiently generate T_RM_ in skin, mucosae and the liver (25, 60, 70). Local antigen expression, and thus the local reactivation of T_RM_ precursor cells by antigen-presenting cells (APC), might also serve the expression of cytokines and chemokines required to guide T_RM_ differentiation and localization (55, 74), which could explain why in some experimental settings antigen is required (75), but not in others (22).

T_RM_ heterogeneity is particularly evident with regard to their expression of adhesion molecules. T_RM_ in different organs (and even further, different subsets of T_RM_) show sometimes combined, sometimes exclusive expression of adhesion molecules such as CD103 (IntegrinαE), CD49a (Integrinα1β1), LFA-1 (IntegrinαLβ2), and E-Cadherin (22, 24, 28, 37, 46, 76, 77). Depending on their interaction partner, adhesion molecule expression on a specific T_RM_ subset probably serves its specific retention and positioning in their tissue of residence (68). CD103 mediates epithelial localization and T_RM_ retention in the skin and gut by interacting with E-Cadherin (4, 22, 69, 78), while CD49a expression anchors T_RM_ to the collagen matrix (79). Besides T_RM_ localization, expression of adhesion molecules has also been linked to T_RM_ functionality. CD103 expression has been associated with an enhanced cytotoxic capacity of CD8+ T cells toward E-Cadherin-expressing target cells (80). Likewise, CD49a expression by human skin T_RM_ seems to discriminate between IFN-γ- and IL-17A-producing cells (24). We are however only beginning to understand how the exposure of T_RM_ precursors to their specific inflammatory context affects T_RM_ differentiation and functionality.

Cytokine redundancy (the common use of receptors and receptor subunits by different cytokines) and pleiotropy (multiple different functions exerted by one cytokine) are possible explanations for some of the observed variations in the dependency of T_RM_ generation on cytokines in different experimental contexts. Interestingly, resting non-activated T cells share a common receptor (CD122/γc) for IL-2 and IL-15. It seems therefore likely that in conditions in which T_RM_ precursors are exposed to e.g., high levels of IL-2 during the acute inflammatory response, IL-15 signaling becomes redundant for T_RM_ generation. As mentioned above, both IL-7 and IL-15 can mediate pro-survival as well as homeostatic proliferation, and a certain functional redundancy might occur between these two cytokines, depending upon which receptors predominate on T_RM_ or their precursors and which cytokine is available in the tissue niche occupied by T_RM_. Consistent with this idea, IL-15 dependency of T_RM_ varies considerably between different organs and might be differentially required for T_RM_ differentiation, survival and/ or homeostatic proliferation (81). This could also explain why expression levels of anti-apoptotic signaling molecules in T_RM_, such as Bcl-2, vary between organs, as do the rates of their spontaneous proliferation (22, 29, 32). Thus, it seems possible that for maintaining a stable T_RM_ population, T_RM_ longevity and potential for self-renewal can partly substitute for each other and the signals driving either process might therefore be functionally redundant to some extent. Similarly, transcriptional programming of T_RM_ precursors might vary between one tissue to another. Hobit and Blimp1 have been described to play a partially redundant role during T_RM_ differentiation, but depending on the tissue, T_RM_ generation is more dependent on one of these transcription factors than the other (59). This indicates that transcriptional regulation of T_RM_ differentiation could be incited in a different manner depending on the tissue niche and inflammatory context, possibly giving rise to T_RM_ of different reactivity and functional potential (Figure 1A). In support of this concept, a recent study describes that the presence of pro-inflammatory cytokines like type I interferons and IL-12 drive differentiation of CD103– T_RM_ (74), in contrast to the TGF-β-dependent differentiation of CD103+ T_RM_ (22, 55, 78).

During their differentiation and long-term maintenance, T_RM_ have to adapt to the metabolic environment of their tissue of residence. In most NLT, nutrients such as glucose and certain amino are more limited than in the circulation, and invading T cells need to adapt their metabolic processes to match their energy demands in this environment (82). While glucose plays a central role as energy source for all T subsets, activated T cells show especially high glycolysis rates and also fuel glucose-derived carbons into anabolic pathways such as fatty acid synthesis (83). Further, T cells are dependent on amino acid uptake and metabolism for full activation and differentiation (84–86). However, memory T cells critically rely on fatty acid oxidation (FAO) as an energy source (87–89), for which they synthesize long-chain fatty acids as substrates from glycolytic intermediates intracellularly (90). By contrast, T_RM_ in the skin and adipose tissue rely on uptake of fatty acids from the extracellular space (91, 92), possibly due to the limited amount of glucose available for *de novo* fatty acid synthesis. However, it remains to be determined if T_RM_ in more nutrient-rich organs such as the gut, liver and brain might show distinct tissue-specific metabolic adaptations.

Despite providing the energy for T cell expansion and survival, the metabolic environment also dictates T cell differentiation and effector function (93). Cytokine production, cytotoxicity, migration, and tissue invasiveness as well as the differentiation of memory T cells are instructed by metabolic changes (87, 94–97). One central regulator of this so-called metabolic reprogramming is mammalian target of rapamycin (mTOR) (98). mTOR is phosphorylated in response to TCR ligation, cytokine signaling as well as intracellular energy state. In turn, mTOR regulates CD8+ T cell differentiation via T-bet and Eomesodermin (99) as well as via the regulation of fatty acid metabolism (87). Inhibition of mTOR leads to a higher number of memory precursors and circulating memory T cells (94), by contrast, formation of long-lived T_RM_ in mucosal tissues is impaired (100). Interestingly, activation of mTOR (together with phosphoinositol-3-kinase) induces downregulation of KLF2 and S1P1 in activated T cells (101), indicating that mTOR activation during T_RM_ differentiation could contribute to establish tissue retention. In line with this, upregulation of CD69 on γδ T cells has been shown to enhance uptake of the amino acid tryptophan, which in turn enhanced mTOR- and arylhydrocarbon receptor (AhR)-dependent signaling pathways (102). AhR has been shown to be required for generation of T_RM_ in the skin (103), further corroborating the idea of a mechanistic link between the metabolic, possibly tissue-specific, environment encountered by T_RM_ precursors and the successful formation of a tissue-resident and long-lived T cell population.

Altogether, it seems likely that the combination of antigen load, inflammatory signals and nutrients in a tissue-specific niche creates a specific environmental context for T_RM_ differentiation and maintenance (Figure 1). Given that some T_RM_ niches, especially mucosal tissues and epithelial layers, undergo constant turnover and replacement of cells, it seems likely that the inflammation-induced T_RM_ niche undergoes certain changes in cellular composition and expression of T_RM_-maintaining factors. To date, the exact sources of these determining factors still remain largely unknown. It might even be possible that the T_RM_-maintaining niche in some organs or under certain circumstances has only a limited lifespan, which could explain why T_RM_ are not maintained long-term in some experimental settings (104). The environmental context probably determines not only the functional features of T_RM_ residency and maintenance but also T_RM_ responsiveness toward new inflammatory stimuli during a secondary infection (see Figure 1A). Future studies are needed to reveal more context-dependent variations in T_RM_ generation and functionality, discovering new targets, potentially in a tissue-specific manner, for experimental and therapeutic manipulation of T_RM_.

## T_RM_ in acute-resolved infection

T_RM_ serve as a front-line defense against viral re-infection in various tissues. Due to their unique positioning, often directly at barrier surfaces, they can rapidly detect invading pathogens and provide immediate immune function. In comparison, immune surveillance by circulating memory T cells is slower and often allows virus spread for several days before sufficient recruitment, local expansion, and differentiation of peripheral memory T cells takes place to confine and successfully combat infection (27, 32). This notion is supported by a breadth of experimental models, that demonstrate accelerated pathogen control in the presence of T_RM_ at the pathogen entry site in comparison to circulating memory T cells alone (9). Protective functions of T_RM_ have been described for barrier tissues such as the skin (4, 27, 70, 105), the lung (106–109), the gut (48), and the reproductive tract (110). T_RM_ localized to body surfaces may thus play an important role to prevent systemic infection by recurring pathogens invading via the skin and mucosae and to limit extensive tissue damage and scarring at the entry sites. As a consequence, T_RM_ of a multitude of epitope specificities accumulate with age at these pathogen entry zones in free-living mice and humans (17, 47, 111). Interestingly, a protective role of T_RM_-mediated immune defense has been described also for internal organs such as the liver and the brain (25, 32), which display unique immune-regulatory functions (112, 113). As such, immune cell activation is impeded in these organs, e.g., due to low expression of MHC molecules, and often occurs with considerable delay, which increases the risk of persistent and widespread infection. The latter in turn can contribute to more severe immunopathology once an immune response is finally triggered. Similar to their positioning at epithelial surfaces in barrier tissues, T_RM_ in the brain and liver are also preferentially located at potential pathogen entry sites, be it in meninges and close to brain blood vessels (32) or liver sinusoids (25). This enables T_RM_ to quickly react and eliminate invading pathogens and thereby protect these vulnerable organs from potentially harmful inflammation.

Upon re-encounter of their cognate antigen, T_RM_ employ two main paths to assure protection against the recurring pathogen. Firstly, they instantly provide highly potent cytotoxic effector functions that can eliminate the initially infected cells (barrier immunity) (27, 32). Indeed, a subset of T_RM_ constitutively expresses Granzyme B, and perforin-mediated elimination of infected cells contributes to their protective effect in the brain (32). Secondly, T_RM_ trigger a variety of local and recruited innate and adaptive immune mechanisms that can even provide bystander resistance to unrelated pathogens (39, 105, 110). T_RM_-derived interferon-γ (IFN-γ) plays an important role by stimulating the expression of adhesion molecules and chemokines that facilitate endothelial transgression of peripheral memory T and B cells (39). Further, the expression of IFN-γ-responsive genes—many of them with direct anti-viral functions—in uninfected bystander cells limits pathogen spread (105). Moreover, Granzyme B can deactivate a viral protein in neurons during latent HSV infection without inducing neuronal apoptosis (114) and IFN-γ can even purge viruses from infected cells in a non-cytolytic manner, a process that seems important to maintain tissue homeostasis in non-regenerative tissues such as the brain (115, 116). It is important to note that the protective capacity of T_RM_ related to their cytotoxic activity and cytokine production requires the presentation of cognate antigen on MHC-I molecules, even though T_RM_ can show signs of bystander activation in an inflammatory environment (32).

The protective capacity of T_RM_ makes their generation a new objective for the development of vaccines. Indeed, skin vaccination and scarification during small pox vaccination that has now been associated with the generation of T_RM_ has been shown to provide superior protective immunity than hypodermal injection (117). Alternatively, the above-mentioned “prime and pull” and “prime and trap” vaccination strategies, in which systemic administration of a vaccine is combined with local application of chemokines or antigen, improves immunological barrier functions through T_RM_ generation (25, 60, 70). Interestingly, upon recruitment and activation in skin and mucosae, some T cells exit and give rise to SLO-associated T_RM_ (118). Being positioned at entry sites for draining peripheral antigen, these SLO T_RM_ provide a second line of defense and extend T_RM_-mediated immune memory to the draining lymphoid tissue (119). During antigenic re-challenge, T_RM_ are the predominant population undergoing secondary expansion and together with recruited circulating T cells give rise to new generations of T_RM_ (120, 121). This implies that protective immunity mediated by T_RM_ can be boosted by repeated local immunizations. Further, infections with different pathogens can lead to a persisting T_RM_ population that contains multiple specificities at once, which provide broader and more efficient protection (122). Future vaccination approaches implementing these new insights could thus improve T-cell-mediated protection at external and internal anatomical barrier sites.

## T_RM_ and chronic inflammation

Chronic inflammation results from repeated or continuous immune cell activation by recurrent or persisting antigens. Such responses are desirable to control latent-reactivating or persistent infections and to eliminate neoplastic cells. However, aberrant inflammation caused by environmental or self-antigens carries the risk of developing chronic inflammatory diseases, such as allergies and autoimmune diseases (AD). Indeed, T_RM_ have been detected in several human inflammatory diseases (10, 123) (see Table 2). In principle, two main roles for T_RM_ in chronic inflammatory settings can be envisaged. T_RM_ can be drivers of chronic inflammation, thereby providing a compartmentalization of the immune response. And in a not necessarily exclusive scenario, T_RM_ could trigger the bystander activation of allergen-reactive or self-reactive T cells and thereby serve as contributing triggers to chronic inflammatory diseases.

**Table 2:** T_RM_ in human chronic inflammatory diseases.

**Diseases**	**Phenotype**	**References**
Allergic contact dermatitis	CD3+	(58)
DED	CCR7– CD45RO+/– CD69+ CD103+/–	(124)
Chronic rhinosinusitis	CD69+ S1P1–	(125)
FDE	CD69+ GrB+; CD45RA+ CD62L–CCR7– CD103+	(126, 127)
Psoriaris	CD103+; CD103+/– CD45RO+; CD103+ CD49a+ GrB+	(128–131)
Systemic sclerosis	CD69+ CD103+/–	(132)
Type I diabetes	CD69+ CD103+; CD69+ CD103+/–	(133, 134)
Multiple sclerosis	CD69+ CD103+/– GrB+/– S1P1–	(135)
HIV-1	CD69+ CD103+/– S1P1–	(136)
HBV	CD69+ CD103+/–; CD69+ CD103+/– GrB+/–	(137, 138)
HCV	CD69+ CD103+/– GrB+/–	(138)
Chronic pancreatitis	CD103+	(139)
Rasmussen's encephalitis	CD103+	(140)
HSV-2	CD69+ CD103+/–	(141)
EBV	CD103+	(142)
Breast cancer	CD69+ CD103+ GrB+	(143)
Lung cancer	CD62L– CD69+ CD103+; CCR7– CD62L– CD69+ CD103+ CD49a+ S1P1–	(144, 145)
Ovarian cancer	CD103+/–	(146)
Colorectal cancer	CD69+ CD103+/– CD49a+/–	(147)

*DED, dry eye disease; FDE, fixed drug eruption; HIV-1, human immunodeficiency virus-1; HBV, chronic Hepatitis B virus; HCV, chronic hepatitis C virus; HSV-2, herpes simplex virus-2; EBV, Epstein-Barr virus*.

### T_RM_ functionality in the context of persisting antigen

#### T_RM_ in chronic infection

One of the earliest reports on resident T cell responses came from latently-infected sensory ganglia, in which HSV reactivation was controlled by a non-circulating T cell population (148, 149). Together with the above-mentioned observations during prime-and-boost vaccinations (122), this demonstrates that T_RM_ may retain their inflammatory activity over repeated rounds of antigen stimulation. In the best scenario, this will prevent virus reactivation and ensure continuous virus latency and limitation of virus spread. Indeed, T_RM_ can be detected in sanctuaries of persistent viruses such as human and mouse Cytomegalovirus (CMV) (150, 151), Hepatitis B virus (HBV) (67), Hepatitis C virus (HCV) (138), and Human Immunodeficiency Virus (HIV) (136, 152). Interestingly, high T_RM_ numbers in HBV-infected liver and HIV-infected gut as well as clonal expansion of SLO T_RM_ have been inversely correlated to virus loads and associated with spontaneous resolution of chronic infection (67, 136, 152), provoking interest in T_RM_-directed therapeutic approaches (137). Infection with most persisting viruses leads to chronic immune activation over time, including accumulation of a large virus-specific T cell population, a process referred to as “memory inflation” (153). Inflationary T cells can acquire a T_RM_-like phenotype and become resident, e.g., in the salivary gland, despite being probably an ontogenically-different T cell subset (154). Chronic inflammatory tissue damage is the common long-term consequence of persisting virus infection. Since HBV-specific T_RM_ overcome immunosuppressive mechanisms in the liver and have high expression of pro-inflammatory cytokines like IL-2, IFN-γ, and TNF-α (67), it remains possible that T_RM_ are also drivers of tissue damage in the context of chronic virus infections. Therefore, a potential harmful role of T_RM_ in persisting infections merits further investigations, especially at the chronic stage of HBV and HCV infection.

So far, we understand very little about how and whether functional T_RM_ can be generated in conditions in which their cognate antigen is continuously present. Chronic high levels of antigen in some persistent infections, such as Lymphocytic choriomeningitis virus (LCMV) clone 13 or latent CMV, seem to hamper *de novo* generation of T_RM_ (69, 151). It is therefore likely that virus levels have to significantly contract after initial infection to allow for efficient T_RM_ generation, even in the context of chronic infection. Interestingly, when T_RM_ retention is impaired by lack of TGF-β signaling during chronic LCMV clone 13 infection in the gut, a stable population of anti-viral CD8+ T cells is maintained by continuous recruitment (78), indicating that impaired T_RM_ formation can be compensated for. This suggests that depending on the cytokine milieu present during chronic infection, the local T cell pool might consist of variable proportions of T_RM_ and recruited T cells.

#### T_RM_ and cancer

Tumors can be a source of neo-antigens stimulating anti-tumor CD8+ T cell responses (155, 156) and T cell infiltration is a prognostic marker for a beneficial outcome in some cancers (157, 158). Recent studies demonstrate, that a subset of tumor-infiltrating lymphocytes (TIL) in solid tumors resemble bona fide T_RM_ and are associated with its epithelial layers (159–161). T_RM_-like TILs, in particular when they express CD103, have been associated with better prognosis (143–145, 162), a fact that could be explained by an enhanced cytotoxic efficiency upon interaction of CD103 on T_RM_ with its ligand E-Cadherin on tumor cells (80). Accordingly, experimental strategies inducing tumor-specific T_RM_ show superior tumor control in comparison to approaches that solely generate circulating tumor-specific effector T cells (163–165).

Tumor cells rely heavily on the uptake and metabolism of glucose and other nutrients, resulting in a metabolically-deprived tumor microenvironment (TME) (166, 167). Tumor-infiltrating T lymphocytes (TIL) are further subject of active immunosuppression by myeloid-derived suppressor cells (MDSC) and regulatory T cells (168). MDSC express ligands for immune checkpoint inhibitors (e.g., PD-L1 and PD-L2) and can also contribute to nutrient deprivation in the TME by uptake and metabolism of arginine. As a consequence of the increase of lactate in the TME, TIL lose cytotoxic effector functions and show impaired motility (169). T_RM_ adapt to the metabolic environment of their tissue of residence by utilizing free fatty acids (92), and are under certain circumstances resistant to checkpoint blockade (67). This indicates that tumor-specific T_RM_ might be better adapted to the immunosuppressive tumor microenvironment than their circulating counterparts (8). This opens new avenues for cancer immunotherapies. T_RM_ already present in the tumor could be functionally enhanced by checkpoint inhibitors, potentially together with increasing their catabolic fatty acid metabolism. Indeed, administration of a PPAR-α agonist or free fatty acids increases the functionality of TIL in a melanoma model, especially in combination with anti-PD-1 treatment (170). Moreover, one could envisage to genetically engineer T cells for cell therapy with the aim to promote T_RM_ generation. Recently, such an approach has been realized by modifying chimeric antigen receptor (CAR) T cells to express orthogonal IL-2 receptors allowing for a specific targeting of the transferred cell population (171). A better understanding of T_RM_ differentiation and maintenance could inform a similar strategy aiming at increasing T_RM_ differentiation, maintenance of functionality during CAR T cell therapy. In addition, reprogramming of tumor-infiltrating dendritic cells with β-glucan curdlan in a humanized mouse model of breast cancer enhances the differentiation of CD103+ TIL via DC-derived TGF-β production, resulting in rejection of an established tumor (172), highlighting how adoptive cell therapy can target T_RM_ differentiation.

It remains unknown, how tumor-associated T_RM_ are generated. Analogous to persistent infections (69), tumor-specific T_RM_ are chronically exposed to their cognate antigen, which could impair their successful differentiation. Otherwise, one may speculate that tumor-associated T_RM_ are already generated after development of the first tumor cells when there is still little cognate antigen present. This would imply that these T_RM_ could also play a role in the control of tumor transformation, since they might co-reside with a primary tumor for years. Therapeutic induction of tumor-specific T_RM_, together with other resident populations, could enforce local anti-tumor immune response to the cancer (173) and may help to eradicate tumor cells from the body as well as reduce systemic side effects. To take advantage of this therapeutically, application of e.g., viral vectors that efficiently generate local T_RM_ with only limited numbers of peripheral tumor-specific effector cells could be envisaged. The constitutive expression of checkpoint inhibitors by T_RM_ (19) also harbors the hope that the anti-tumor activity of endogenous or therapeutically-induced T_RM_ could be further enhanced by checkpoint inhibitor blockade (174). However, one has to keep in mind, that enhancing T_RM_ activity and expansion might come with undesired side effects, and disinhibiting T_RM_ might in turn give rise to tumors. Due to their localization and non-circulating behavior, T_RM_ are refractory to most immune ablative therapies, as evidenced by mycosis fungoides, a human T_RM_-like skin lymphoma (42).

#### T_RM_-driven chronic inflammatory diseases

T cells specific for self-antigens or environmental antigens are considered active drivers of diverse allergic reactions such as food and drug allergies, asthma, and diabetes, as well as autoimmune diseases such as psoriasis, inflammatory bowel disease, and multiple sclerosis (MS). In the past, these diseases were considered to be driven by effector or effector memory T cells that infiltrate the affected organ, however, several lines of evidence suggest that some of these chronic inflammatory diseases or some disease stages are instead predominantly driven by resident immune cells (10, 123). Therapeutic inefficacy of drugs inhibiting T cell recruitment indicates a putative T_RM_ involvement in disease progression since T_RM_ reside behind the blood-tissue barrier and are often refractory to systemic blockade or ablation. This applies, for example, to psoriasis (128) and progressive stages of MS (135). Interestingly, new exacerbations in fixed drug eruption (FDE) and psoriasis frequently occur at sites of previously-resolved skin lesions, indicative of an involvement of localized immune memory (175). Likewise, so-called smoldering lesions characterized by activated macrophages/microglia together with T cells at their fringes are almost exclusively observed in progressive MS (176, 177). Evidence of T_RM_ persistence has been found in resolved psoriatic skin lesions (178) and in chronic MS lesions (135), in which they constitute the dominant T cell subset (our own unpublished observations). Indeed, psoriatic normal-appearing skin contains all immune components necessary to elicit lesion formation upon an environmental trigger (44) and compartmentalized inflammation has been correlated to cumulative brain damage during progressive MS. Beyond that, indications for T_RM_ involvement were found in many other chronic inflammatory diseases (see Table 2), suggesting possible common mechanisms for their development and involvement in disease pathogenesis.

One hallmark of allergic exacerbations is the short time frame (24–48 h) after exposure to the environmental trigger, in which exacerbations occur as is observed in FDE, a T cell-driven allergic local cutaneous reaction to certain administered drugs (179). This short time interval between trigger and reaction cannot be explained by recruitment of circulating T cells, which typically takes longer (58). Evidently, analog time intervals are difficult to determine for flares of autoimmune diseases, where the trigger of an exacerbation is unknown. T_RM_ generally have a low threshold for re-activation, and presence of their cognate antigen (e.g., by exogenous administration of peptide) is usually enough to elicit expression of cytolytic effector molecules and cytokines (39, 105, 110, 180). Likewise, induction of antigen expression in non-hematopoietic cells, without concomitant danger signals, is sufficient to elicit activation of resident T cells in the skin (180), probably via intermediary presentation of antigen on professional APC (180, 181). One may speculate that the reactivation threshold of T_RM_ depends upon the residence organ and/or the T_RM_-generating stimulus, which could in turn result in organ-dependent susceptibility to allergic and self-reactive T_RM_ responses (see Figure 1B).

Allergen- or self-reactive T cell responses are usually considered to be elicited by preceding sensitizing events, which are hypothesized to occur in two main ways. Sequence similarities between a pathogen and an allergen- or self-antigen (also called molecular mimicry) can elicit pathogen-specific T cells that may cross-react to allergens or self-antigen upon future exposure (182). In case these T cells differentiate into T_RM_, a compartmentalized allergic or autoreactive T cell response is the consequence. Alternatively, exposure of allergen- or self-reactive T cells to a pro-inflammatory environment, such as that generated during an infection, could elicit their bystander activation and recruitment (183). As mentioned above, non-specific inflammation can be sufficient to allow for T_RM_ differentiation in the absence of their cognate antigen (25, 60, 69, 70), making this a plausible scenario for the generation of T_RM_ specific for environmental or self-antigens. Moreover, bystander activation could also explain how T_RM_ could serve as triggers for chronic inflammatory diseases. Antigenic challenge of T_RM_ not only induces a new generation from the pre-existing T_RM_ pool, but can also activate and recruit bystander T cells of unrelated antigen-specificity, which give rise to newly formed T_RM_ (120, 121). Accumulation of T_RM_ can further lead to the displacement of other pre-existing resident immune cells (47, 103). Although this might serve to replace the more resident innate immune system by a more specific and efficient resident adaptive immune system, it carries the risk of replacing mostly “naive” immune cells by more trained, and possibly more pro-inflammatory immune cell components. Indeed, T_RM_ are often observed in clusters together with mature professional APCs and often CD4+ T helper cells as well as other immune cells, indicative of organ-associated lymphoid tissues (19, 29, 64, 184, 185). These specific niches are speculated to contribute to chronic inflammation, since they provide an optimal environment for T cell re-stimulation (186–188). Such structures have been shown to contribute to T_RM_ maintenance by chemokine and cytokine production (64, 184), however, whether T_RM_ actively sustain these immune cell clusters is not clear.

Altogether, this supports the idea that pathogen-specific T_RM_ generated during an infection could trigger and/or drive chronic inflammatory diseases. A possible connection between T_RM_ and chronic inflammation could also provide a mechanistic explanation for the observed epidemiological association of infections and the development or exacerbation of allergic and autoimmune diseases (189). In many chronic inflammatory diseases, not only CD8+ T_RM_, but also other immune cells such as T helper (Th) cell subsets, regulatory T cells, APCs and innate lymphocytes can probably become resident and thereby contribute to a compartmentalized immune response that is resistant to many systemic immunomodulatory therapies. Thus, more research efforts are needed to understand the requirements for the differentiation and maintenance of resident immune cells in order to be able to functionally impair or even deplete T_RM_ in chronic inflammatory diseases. By identifying signaling pathways involved in T_RM_ retention and maintenance, we are currently undertaking the first steps toward a specific targeting of T_RM_ without global immunosuppression. One possibility could be to interfere with T_RM_ metabolism. Pharmacological treatments with Rapamycin (100) or inhibitors of FAO such as Trimetazidine and Etomoxir (92) have already been shown to decrease T_RM_ numbers in experimental models.

Immunoregulatory mechanisms are in place to prevent extensive T_RM_ accumulation in some organs or their over-activation. T_RM_ generation is intimately linked to TGF-β (55, 78), a cytokine associated to resolution of infection. This indicates that T_RM_ differentiation might not occur in presence of chronic antigen exposure, thereby preventing extensive T_RM_ generation in chronic inflammatory settings. Further, T_RM_ can express inhibitory receptors, such as PD-1, Lag3, and Tim3 (19, 29, 49, 107), in principle making them susceptible to checkpoint inhibition. Although T_RM_ have the possibility to overcome PD-1-associated inhibition (67), exhausted T_RM_ have been detected in immune privileged sites such as the eye (190). T_RM_ are also susceptible to regulatory T cell-mediated immunosuppression (191, 192). For the lung, liver and brain and other immune privileged sites, even mechanisms of natural suppression of T_RM_ accumulation have been suggested (104) that could assure tissue homeostasis by prevention of T_RM_ accumulation.

## Conclusion

Vaccine strategies inducing T_RM_ against recurring infections are promising approaches to improve immunological protection. Equally, tumor-specific T_RM_ might help to eradicate aberrant tumor cells from the body and enforce a localization of this response, thereby minimizing systemic side effects. However, several challenges have to be overcome to realize these goals, which are firstly of a technical nature. T_RM_ generation cannot be monitored in peripheral blood and therefore requires the taking of biopsies from target organs, which might not always be easily performed. Further, suitable vaccination vectors need to be designed that allow the efficient local induction of specific T_RM_ and that do not result in unwanted side effects such as bystander-induced self-reactive T_RM_. Until now, most research studies have focused on the overwhelmingly positive role of T_RM_ acting against infected or tumorous cells, however we still lack an appropriate understanding of the possible physiological consequences of T_RM_ persistence. Further research efforts are warranted to better understand the role of T_RM_ in chronic inflammatory diseases in order to identify the risks in amplifying T_RM_ numbers or function. So far, we are lacking appropriate mouse models allowing specific genetic targeting of T_RM_ and are not able to completely deplete already-established T_RM_. It is therefore instrumental to perform detailed preclinical and clinical studies to gain more insight into T_RM_ biology and its adaptation during different experimental regimens and in different tissues to allow for a safe and efficient therapeutic tissue targeting of T_RM_.

## Author contributions

All authors listed have made a substantial, direct and intellectual contribution to the work, and approved it for publication.

### Conflict of interest statement

The authors declare that the research was conducted in the absence of any commercial or financial relationships that could be construed as a potential conflict of interest.
